# Green Tea Epigallocatechin-3-Gallate Regulates Autophagy in Male and Female Reproductive Cancer

**DOI:** 10.3389/fphar.2022.906746

**Published:** 2022-07-04

**Authors:** Sze Wan Hung, Yiran Li, Xiaoyan Chen, Kai On Chu, Yiwei Zhao, Yingyu Liu, Xi Guo, Gene Chi-Wai Man, Chi Chiu Wang

**Affiliations:** ^1^ Department of Obstetrics and Gynaecology, The Faculty of Medicine, Prince of Wales Hospital, The Chinese University of Hong Kong, Hong Kong, China; ^2^ Department of Obstetrics and Gynaecology, Shenzhen Baoan Women’s and Children’s Hospital, Shenzhen University, Shenzhen, China; ^3^ Department of Ophthalmology and Visual Sciences, Hong Kong Eye Hospital, The Chinese University of Hong Kong, Hong Kong, China; ^4^ Department of Obstetrics and Gynecology, School of Medicine, Renji Hospital, Shanghai Jiao Tong University, Shanghai, China; ^5^ Department of Orthopaedics and Traumatology, The Faculty of Medicine, Prince of Wales Hospital, The Chinese University of Hong Kong, Hong Kong, China; ^6^ Li Ka Shing Institute of Health Sciences; School of Biomedical Sciences; and Chinese University of Hong Kong-Sichuan University Joint Laboratory in Reproductive Medicine, Prince of Wales Hospital, The Chinese University of Hong Kong, Hong Kong, China

**Keywords:** green tea, EGCG, autophagy, reproductive cancers, anticancer

## Abstract

With a rich abundance of natural polyphenols, green tea has become one of the most popular and healthiest nonalcoholic beverages being consumed worldwide. Epigallocatechin-3-gallate (EGCG) is the predominant catechin found in green tea, which has been shown to promote numerous health benefits, including metabolic regulation, antioxidant, anti-inflammatory, and anticancer. Clinical studies have also shown the inhibitory effects of EGCG on cancers of the male and female reproductive system, including ovarian, cervical, endometrial, breast, testicular, and prostate cancers. Autophagy is a natural, self-degradation process that serves important functions in both tumor suppression and tumor cell survival. Naturally derived products have the potential to be an effective and safe alternative in balancing autophagy and maintaining homeostasis during tumor development. Although EGCG has been shown to play a critical role in the suppression of multiple cancers, its role as autophagy modulator in cancers of the male and female reproductive system remains to be fully discussed. Herein, we aim to provide an overview of the current knowledge of EGCG in targeting autophagy and its related signaling mechanism in reproductive cancers. Effects of EGCG on regulating autophagy toward reproductive cancers as a single therapy or cotreatment with other chemotherapies will be reviewed and compared. Additionally, the underlying mechanisms and crosstalk of EGCG between autophagy and other cellular processes, such as reactive oxidative stress, ER stress, angiogenesis, and apoptosis, will be summarized. The present review will help to shed light on the significance of green tea as a potential therapeutic treatment for reproductive cancers through regulating autophagy.

## Introduction

Male and female reproductive cancers remain one of the biggest health concerns worldwide due to the high prevalence, yet therapeutic management remains a great challenge. A low survival rate reflects the lack of efficient treatments and risks of recurrence (Garcia-Aranda and Redondo, 2019; Hanna et al., 2020; Hawsawi et al., 2020). Current treatments for reproductive cancers bring fertility-related side effects. The risk of infertility associated with gynecologic malignancies is high, with a successful pregnancy rate of only above 40% (Poorvu et al., 2019). Hysterectomy is one of the most common management of ovarian and endometrial cancers, removing the critical reproductive organs. In breast cancer, a negative impact on the reproductive system has been reported after chemotherapy (Hickey et al., 2009). Radiation therapy that is directed to the reproductive organs disturbs hormone production and imposes fertility risk. Adjuvant therapy for testicular cancer affects fecundity (Kim et al., 2010; Poorvu et al., 2019). Recently, modulation of autophagy is proposed as a novel approach to tackle cancer. In this review article, we will report the therapeutic effects of green tea, particularly EGCG, on female and male reproductive cancers and its possible mechanisms behind it. In addition, this review will emphasize the crosstalk of EGCG between its regulated anti-carcinogenic properties with autophagy on highlighting its role as an autophagy modulator toward reproductive cancer. Importantly, we hope to provide a perspective on green tea catechins and EGCG as potential autophagy modulator for the treatment of reproductive cancer.

### Incidence, Mortality, and Prevalence of Reproductive Cancer

Cancer is a major leading burden of disease worldwide, accounting for nearly 10 million deaths in 2020, or nearly one in six deaths. In 2050, an estimated 6.9 million new cancers will be diagnosed in adults aged 80 years or older worldwide (20.5% of all cancer cases) (Pilleron et al., 2021). Among both male and female, reproductive cancer contributes to a high incidence and mortality on the causes of death worldwide (Garcia-Aranda and Redondo, 2019; Hawsawi et al., 2020). The following cancers are exclusive to the female reproductive system including cancer originating at the ovary (ovarian cancer), cervix uteri (cervical cancer), and corpus uteri (endometrial cancer). While these cancers are exclusive to the male reproductive system, these include cancer originating at the prostate gland (prostate cancer) and the testis (testicular cancer). As for breast cancer, this can affect both males and females.

Ovarian cancer cells arise from epithelial, stromal, or germ cells of the ovary (Horowitz et al., 2020). The cells disseminate through the perineal cavity and metastasize to the omentum (Yeung et al., 2015). On the first hand, cancer cells from primary tumor cells can passively be disseminated to a newly established site, with enhanced cancer cells adhesion, invasiveness, motility, and a supportive microenvironment. On the other hand, it can undergo a hematogenous metastatic mechanism *via* epithelial-mesenchymal transition (Yeung et al., 2015). In 2020, ovarian cancer accounts for 313,959 new cases, and 207,252 deaths. The crude death rate of ovarian cancer was 5.4 per 100,000 female population. The age-standardized death rate of ovarian cancer was 4.2 per 100,000 standard population (WHO, 2022). Diagnosis of ovarian cancer is often delayed; it could be more treatable if it is diagnosed earlier (Evans et al., 2007).

For cervical cancer, it has been widely accepted that Human papillomavirus (HPV) infection is an attributable factor (Walboomers et al., 1999). HPV oncoproteins E6 and E7 block p53 and retinoblastoma protein, which function as tumor suppressors, as well as engage in multi-step carcinogenesis such as viral DNA replication and proliferation, leading to invasive cervical cancer development (Walboomers et al., 1999; Yugawa and Kiyono, 2009; Den Boon et al., 2015; Gupta and Mania-Pramanik, 2019). Worldwide, cervical cancer is reportedly the fourth most incident cancer and the fourth leading cause of cancer-related death, with 604,127 new cases and 341,831 deaths in women in 2020. Early on, typically no symptoms are seen. Later, symptoms may include abnormal vaginal bleeding, pelvic pain, or pain during sexual intercourse (WHO, 2022).

Endometrial cancer can be endometrioid (type 1), arising from excess stimulation of estrogen and atypical glandular hyperplasia; or non-endometrioid (type 2) (Lu and Broaddus, 2020; Terzic et al., 2021). Type 1 tumors are associated with significantly enhanced oncogene mutations and tumor suppressor genes; hence, it resembles proliferative endometrium (Hecht and Mutter, 2006; Morice et al., 2016). Type 2 tumors are mainly associated with enhanced TP53 and HER2/neu mutations (Morice et al., 2016; Terzic et al., 2021). In 2020, endometrial cancers newly occurred in 417,367 women and caused 97,370 deaths. This makes it the third most common cause of death in cancers which only affect women, behind cervical and ovarian cancer. Endometrial cancer arises from the endometrium (the lining of the uterus or womb) and is associated with excessive estrogen exposure, high blood pressure, and diabetes. And often, regular screening in those at normal risk is not common.

Breast cancer originates within the inner lining of ducts or the lobules, then disseminates to lymph nodes or other nearby sites. The major cause of mortalities includes metastasis with enhanced cells, or with dysregulated immune responses (Zheng et al., 2017). In 2020, breast cancer is the most diagnosed cancer, with 2.3 million cases and 685,000 deaths globally (Sung et al., 2021). It affects 1 in 7 (14%) women worldwide, making it the world’s most prevalent cancer (Balasubramanian et al., 2019). Breast cancer occurs in every country of the world in women at any age after puberty but with increasing rates in later life (WHO, 2021).

Genetic and environmental factors contribute to the development of testicular germ cell tumors, supported by ethical and geographic evidence (Al-Obaidy et al., 2020). It is also reported that isochromosome 12p alternation is especially associated with the progression and invasiveness of the tumors; and it can be related to abnormalities in malignant transformation, oxidative damage, and DNA repairing (Reuter, 2005; Cheng et al., 2017; Al-Obaidy et al., 2020). Globally, testicular cancer affected about 74,458 new cases in 2020, which resulted in 9,334 deaths in 2020. As found, rates are lower in the developing than the developed world. Onset most commonly occurs in males 20–34 years old, rarely before 15 years old (Hayes-Lattin and Nichols, 2009). Most often, treatment outcomes are better when the disease remains localized.

Prostate cancer development can be *via* intraepithelial neoplasia which is the hyperplasia of luminal cells and progressive loss of basal cells. On the other hand, it is also reported as an androgen-dependent adenocarcinoma, that is characterized by the strong phenotype of lumina cells but without basal cells. In contrast, it can also be castration-resistant and independent of androgens (Testa et al., 2019; Tong, 2021). Prostate cancer is the fourth most incident cancer worldwide, with 1,414,259 new cases and 375,304 deaths in 2020. In males, it was the second most common cancer and had the highest prevalence in 2020. Factors that increase the risk of prostate cancer include older age, family history, and race. About 99% of cases occur after age 50 (Rawla, 2019).

Overall, reproductive cancers comprised 28.11% of incidence, 17.34% of mortality, and 38.05% of prevalence (5 years) of all cancers in the world in 2020, demonstrating its relevance as a public health problem (Table 1). The molecular mechanism of reproductive related cancers had been extensively reviewed, which is a complex carcinogenesis process accompanied by related pathophysiology (Zheng et al., 2017; Gupta and Mania-Pramanik, 2019; Testa et al., 2019; Al-Obaidy et al., 2020; Horowitz et al., 2020; Terzic et al., 2021). These include enhanced growth factors and oncogenes but resisting apoptotic factors and tumor suppressors, as a result of enhanced invasiveness and metastasis. These processes are regulated by different signal transduction systems such as the cell cycle and DNA repair system (Torgovnick and Schumacher, 2015; Li et al., 2020). Noteworthily, there is a rapid growth of research that focuses on the autophagy roles in cancer pathophysiology. Our team previously conducted a comprehensive and systematic analysis on the roles of autophagy in cervical, endometrial, and ovarian cancer progression and chemoresistance (Tam et al., 2021). We agreed with the argumentative roles of autophagy, which are inconsistent with those proposed in previous cancer studies (Janku et al., 2011; Mowers et al., 2017; Lim and Murthy, 2020). In this review, we extend the research to explore autophagy modulators in reproductive cancer.

**TABLE 1:** Incidence, mortality, and prevalence of male and female reproductive cancers worldwide in 2020.

Cancer	Prevalence, 5 years (total cases of all cancers: 44,091,402)			Mortality (total deaths of all cancers: 9,894,402)			Incidence (total new cases of all cancers: 18, 094,716)			Proportion mortality/incidence
	Prevalence in cancer occurrence	Number of cases	%	Cause of cancer-related death	Number of cases	%	Cancer most frequently diagnosed	Number of cases	%	
Breast	1st	7,790,717	17.67	5th	684,996	6.92	1st	2,261,419	12.50	0.30
Cervical	9th	1,495,211	3.39	9th	341,831	3.45	7th	604,127	3.34	0.57
Endometrial	10th	1,415,213	3.21	19th	97,370	0.98	15th	417,367	2.31	0.23
Ovarian	17th	823,315	1.87	14th	207,252	2.09	18th	313,959	1.74	0.66
Prostate	3rd	4,956,901	11.24	8th	375,304	3.79	4th	1,414,259	7.82	0.27
Testicular	23rd	296,686	0.67	32nd	9,334	0.09	27th	74,458	0.41	0.13
Total	—	16,778,043	38.05	—	1,716,087	17.34	—	5,085,589	28.11	—

Information collected from data published at https://gco.iarc.fr/today, accessed on 21st March 2022

## The Role of Autophagy in Reproductive Cancer

In normal physiological conditions, autophagy typically would be maintained at a minimal level of activity. Mainly, cells utilize basal levels of autophagy to aid in the maintenance of biological function, homeostasis, quality-control of cell contents, and elimination of old proteins and damaged organelles for cell survival (Levine and Yuan, 2005; Mizushima et al., 2008). Autophagy happens in lysosome which has a membrane to limit the leakage of the degradative enzyme. There are micro- and macro-autophagy. The former engulfs the cytosolic compound directly by lysosomes, and the latter involves the sequestration of cargoes within an autophagosome, which is a double membrane cytosolic vesicle formed from a phagophore. Macro-autophagy can be specific and targets organelles or invasive microbes, as well as can be nonspecific. The degradation process of autophagosome happens in autolysosome and macromolecules will be released back into the cytosol (Levine and Kroemer, 2008; Mizushima et al., 2008; Mizushima and Levine, 2020). Additionally, the regulation of autophagy can modulate cancer cells’ growth or death.

During the initiation stage of autophagy, phagophore assembly site (PAS) is formed, along with UNC51-like kinase (ULK)1/2 and autophagy-related protein (ATG)13 which translocate to the endoplasmic reticulum to facilitate the formation of class III PI3K-Beclin1-VPS34 complex as phagophore. Phagophore undergoes elongation to form autophagosome, which requires two ubiquitin-like conjugate systems: ATG12-ATG5-ATG16 multimeric system and Phosphatidylethanolamine (PE) to microtubule-associated protein 1 light chain (LC)3 conjugate system. ATG7 and ATG4 facilitate the binding of LC3-PE, which are essential for the expansion of the autophagosome membrane to recognize autophagic cargos. During the maturation stage, the cargo sequestration is completed. The resulting autophagosome fuses with a lysosome to form an autolysosome, meanwhile, lysosomal enzyme degrades the autophagosome contents. At last, the cargo is broken down and released into the cytosol. The lysosome is recycled during the degradation stage (Levine and Kroemer, 2008; Mizushima et al., 2008; Feng et al., 2014; Kaur and Debnath, 2015; Mizushima and Levine, 2020). Based on our previous findings, we reported that several autophagy regulators, consisting of ULK1, Beclin1, ATG5, p62, and LC-3BII, were related to the growth of cervical, endometrial, and ovarian cancer (Tam et al., 2021). Similarly, these autophagy regulators, including Beclin1 and LC-3B, were also shown to modulate prostate adenocarcinoma and breast cancer (Naponelli et al., 2015; Tyutyunyk-Massey and Gewirtz, 2020) while p62, LC3B, and Beclin1 played critical rolesin the survival of testicular cancer (Nakkas et al., 2021). Therefore, by targeting these autophagy regulatory factors directly or indirectly, it could potentially be used to inhibit the progression of reproductive cancer tumors.

### Promotion of Cancer Growth

Autophagy is a physiological cellular process for the degradation and elimination of misfolded proteins and damaged organelles that function in adaptation to starvation, development, cell death, and tumor suppression (Klionsky et al., 2021). This process allows unneeded proteins to be degraded and the amino acids recycled for the synthesis of proteins that are essential for survival. Toward cancer survival, autophagy plays dual roles in contributing to the growth of cancer as well as tumor suppression (Klionsky et al., 2021). Such that, the growth of cancer can be promoted by maintaining the survival of tumor cells that have been starved, or to degrade apoptotic mediators, through autophagy (Su et al., 2013). This can be induced by cytotoxic and metabolic stresses, including hypoxia and nutrient deprivation, to activate autophagy for recycling of ATP and to maintain cellular biosynthesis and survival, which promotes HIF-1α-dependent and -independent activation (Semenza, 2010). This activation would increase the expression of angiogenic factors, such as vascular endothelial growth factor (VEGF), platelet-derived growth factor, and nitric oxide synthase. Also, the induction of autophagy by miRNA-4673 has been found to improve the resistance of cancer cells to radiation (Dokumcu et al., 2018). In addition, suppression of autophagy by deletion of Beclin 1 enhances cell death (Degenhardt and White, 2006; White and Dipaola, 2009). Autophagy presents the cytoprotective ability to cancer cells and favors cell survival by coping with the intracellular and environmental stress. Therefore, potent inhibitors to limit the progression of autophagy can be an alternative strategy (Amaravadi et al., 2019; Mizushima and Levine, 2020), such as Metformin in prostate cancer (Farrow et al., 2014); Chloroquine in prostate, breast, and endometrial cancer (Solomon and Lee, 2009; Farrow et al., 2014; Nunez-Olvera et al., 2019); Bortezomib and HDAC6 inhibitor Panobinostat in breast cancer (Maycotte and Thorburn, 2014); Elaiophyl in ovarian cancer (Zhao et al., 2015) *etc*. Autophagy inhibitors should be used only if cancer cells are more reliant on autophagy than normal cells (Mizushima and Levine, 2020).

### Promotion of Cancer Cell Death

Various studies have shown the regulation of autophagy would contribute to the expression of tumor suppressor proteins or oncogenes (Avalos et al., 2014). The previous study has shown xenografts with a lowered expression of Beclin1, a protein that regulates autophagy, to be tumor-prone (Qu et al., 2003). However, another study demonstrated when Beclin1 was overexpressed, the tumor development was inhibited (Liang et al., 1999). Likewise, autophagy can indirectly prevent the release of proinflammatory HMGB1 by limiting necrosis and chronic inflammation to protect against tumorigenesis (Tang et al., 2010). Autophagy brings cytotoxicity effects to promote tumor cell death, which can be completed through degradation of oncogenic proteins (Janku et al., 2011), or *via* reducing metabolic stress, regulation of oxidative stress, or metastasis during tumorigenesis. DNA would be damaged and lead to genomic instability (Mathew et al., 2009; Mizushima and Levine, 2020). It responds to a range of cellular stresses, including nutrient deprivation, organelle damage, and abnormal protein accumulation. Autophagy inducers may be beneficial when there are excess abnormal macromolecules and organelles (Mizushima and Levine, 2020), for example, Rapamycin in breast cancer (Danko et al., 2021); Trastuzumab in HER2 positive breast cancer (Maycotte and Thorburn, 2014); Atorvastatin in prostate cancer (Farrow et al., 2014); Metformin in cervical, breast and endometrial cancer (Lu G. et al., 2021), *etc*.

As mentioned, some anticancer drugs have been shown to regulate autophagy (Cuomo et al., 2019). However, targeting these autophagy modulators as a therapeutical strategy remains a huge obstacle (Levy et al., 2017; Amaravadi et al., 2019). Different side effects were reported, ranging from diarrhea, and sensitization to chemotherapy and anemia (Buzun et al., 2021). Autophagy inhibitors had also been reported to sensitize immune response in therapy, as well as influence tumor stroma and normal cell function (Gewirtz, 2016; Noguchi et al., 2020). Hence, natural product as autophagy modulator has been proposed, owing to their safe profiles whilst showing high effectiveness to diminish tumor cells (Zhang Q. Y. et al., 2018; Rahman et al., 2020; Al-Bari et al., 2021). Natural product to regulate autophagy is also a novel therapeutic approach in improving fertility (Zhu et al., 2019; Park et al., 2021). In addition, natural products can be used to target multiple pathways, thus providing greater flexibility and potential for a variety of cancer types and stages.

## Green Tea

Tea is brewed from the leaves and buds of the plant *Camellia sinensis*. With extreme popularity particularly in Asia, it is ranked the second most common beverage consumed worldwide (behind water) (Graham, 1992). In general, teas from this plant can be grouped into green, black, or oolong tea. Among the tea consumption, approximately 20% is in the form of green tea. As green tea has not undergone the same withering and oxidation process as black or oolong tea, it contains the highest amounts of natural polyphenols (catechins) (around 5–7 mg/g in green tea; while that in black tea is only around 0–3 mg/g). These catechins account for approximately 30–40% of the extractable solids of dried green tea leaves, alkaloids (such as caffeine and theobromine), carbohydrates, tannins, and minerals (such as fluoride and aluminum) (Kochman et al., 2020). These catechins have been shown to be more powerful antioxidants, both *in vitro* and *in vivo*, when compared to vitamins C and vitamins E (Cabrera et al., 2006; Khan and Mukhtar, 2008; Chacko et al., 2010). The four-major green tea catechins include (−)-epicatechin (EC), (−)-epigallocatechin (EGC), (−)-epicatechin-3-gallate (ECG), and (−)-epigallocatechin-3-gallate (EGCG), which EGCG is the most abundant and biologically active (Schramm, 2013) (Supplementary Figure S1). Green tea has been shown to regulate a range of health issues such as improves digestive and brain function, promotes metabolism, as well as fights and protects against cardiovascular disease, diabetes, cancer and others (Cabrera et al., 2006; Khan and Mukhtar, 2008; Chacko et al., 2010; Kochman et al., 2020). Based on previous studies, the consumption of green tea daily was found to be correlated with a slightly lower risk of death from cardiovascular causes (Xi et al., 2015) and a reduced risk of stroke (Zhang et al., 2015). As EGCG is a powerful antioxidant, it is believed to be an important determinant in the therapeutic qualities of green tea. As the structure of EGCG possessed a gallic acid, the presence of the gallate moiety and the extra phenol ring on EGCG enhance its anticancer property, which traps electrons, as a scavenger of free radicals and reduces oxidative stress (Du et al., 2012; Chu et al., 2017). This powerful antioxidant property of EGCG can suppress neovascularization and regulate vessel permeability, thereby cutting off the blood supply to cancerous cells (Maiti et al., 2003).

### Potential Anticancer Effect of Green Tea

**FIGURE 1:**
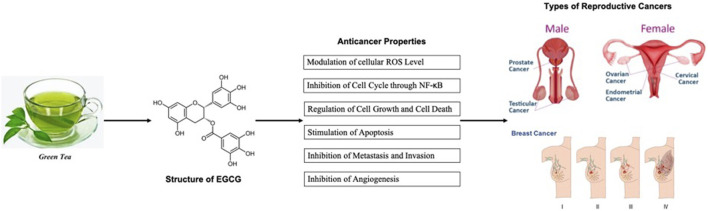
Role of EGCG and green tea catechins on reproductive cancers.

Green tea catechin was shown to target cell cycle regulatory proteins, inhibit cell division, stimulate cell cycle arrest mechanism and promote apoptosis, thus is effective against the progression of the tumor. As green tea catechins, in particular, EGCG, are powerful antioxidants, it is believed they are important determinants in the therapeutic qualities of green tea. As the structure of EGCG possessed a gallic acid, the presence of the gallate moiety and the extra phenol ring on EGCG enhance its anticancer property, which traps electrons, as a scavenger of free radicals and reduces oxidative stress (Du et al., 2012; Chu et al., 2017). This powerful antioxidant property of EGCG can suppress neovascularization and regulating vessel permeability, thereby cutting off the blood supply to cancerous cells (Maiti et al., 2003). EGCG also regulated various transcription factors, protein kinases, and inflammatory cytokines to mediate the invasion, metastasis, and progression of cancerous cells (Shirakami and Shimizu, 2018; Kochman et al., 2020). The summary of the anticancer properties of green tea is shown in Figure 1.

### Experimental Studies

Toward ovarian cancer, experimental studies uncovered the mechanisms of green tea, which are mainly involved in regulating cell proliferation and apoptosis in cells or xenograft models. Green tea targeted CD44 as a safe nanomedicine treatment, thus inhibiting tumor progression and recurrence (Bae et al., 2017). It had been demonstrated that EGCG efficacies *via* targeting a number of genes including proliferative related genes JUN, FADD, NFKB1, HIF1α, and matrix metalloproteinases (MMPs) (Xinqiang et al., 2017), apoptotic related genes Bcl-2, Bax, caspases (Panji et al., 2021a) as well as downregulated PTEN/AKT/mTOR pathway to inhibit proliferation *in vitro* and *in vivo* (Qin et al., 2020). EGCG promoted DNA damage response, reduced DNA synthesis, and enhanced oxidative stress to induce apoptosis in cells (Mazumder et al., 2012) (Rao and Pagidas, 2010) (Chan et al., 2006).

Towards cervical cancer, green tea extract was reported to suppress TGF-β induced epithelial–mesenchymal transition *via* reactive oxygen species (ROS) generation, as well as inhibited proliferation and induced apoptosis *via* VEGF, NF-KB, and Akt pathways in cell lines or mice with HeLa xenografts (Singh et al., 2011; Panji et al., 2021b; Khojaste and Ahmadizadeh, 2021). EGCG reduced DNMT to regulate DNA hypomethylation (Khan et al., 2015), induced cell cycle arrest (Ahn et al., 2003), and downregulated p53 expression to regulate proliferation and apoptosis (Muthusami et al., 2013). EGCG inhibited hypoxia-induced HIF-1α to inhibit VEGF, thus regulating angiogenesis (Zhang et al., 2006). Toward endometrial cancer, the effect of the prodrug of EGCG (ProEGCG) exhibited anti-proliferative, pro-apoptotic, and anti-tumor actions *via* ERK/JNK, Akt and PI3K/AKT/mTOR/HIF1α signaling pathway (Wang Q. et al., 2018; Man et al., 2020).

Toward breast cancer, the anti-proliferative and pro-apoptotic effects of green tea catechins were achieved *via* inhibiting VEGF transcription (Sartippour et al., 2002), increasing Bax/Bcl-2 ratio (Li W. et al., 2016) or inducing p27 (kip1) CK1(Kavanagh et al., 2001), cell cycle arrest (Liu et al., 2017), caspases (Mbuthia et al., 2017) and p53 (Santos et al., 2021). In particular, EGCG mediated multiple signaling pathways to inhibit cell proliferation, migration, and invasion, thus limiting tumor progression, which included PI3K, Akt, NF-κB, MAPK, Wnt (Pianetti et al., 2002; Pan et al., 2007) and estrogen receptor (Belguise et al., 2007; Li et al., 2010) signaling pathways. Additionally, EGCG served as an antiangiogenic inhibitor that downregulated the VEGF pathway (Sartippour et al., 2002; Braicu et al., 2013; Crew et al., 2015), as well as an immune modulator that targeted IL-6 and TNF-α (Jang et al., 2013). Several studies also revealed the anti-invasive ability of catechins through mediating MMPs(Roomi et al., 2005a), VASP(Zhang et al., 2009), FOXO3a (Belguise et al., 2007), or p53 (Santos et al., 2021) expressions. In addition to acting as a chemo-preventive agent, green tea catechins also reversed chemotherapy resistance (Esmaeili, 2016; Tuntiteerawit et al., 2020).

While in testicular cancer, *in vitro* study revealed the potential of green tea extract to inhibit MMP and regulated invasion (Roomi et al., 2007). Similarly, studies on prostate cancer showed that green tea catechins suppressed cell proliferation and induced apoptosis by an increased Bax/Bcl-2 ratio (Yang et al., 2019) and *via* regulating the cell cycle (Wang et al., 2012), PI3K, Akt and Erk pathways (Siddiqui et al., 2004). Furthermore, green tea catechins suppressed invasion and migration by TIMPs and MMPs(Deb et al., 2019; Fan et al., 2021); inhibited tumor angiogenesis by downregulating IGF-1 (Harper et al., 2007), HIF1α (Thomas and Kim, 2005) and VEGF (Roomi et al., 2015b); and targets NK-κB (Saedmocheshi et al., 2019), COX-2 (Adhami et al., 2007), interleukin family (Mukherjee et al., 2014), androgen receptor (Stanislawska et al., 2019) to suppress prostate cancer cell growth. A summary of studies based on animal models about the anti-cancer effects of EGCG and green tea catechins on reproductive cancers can be found in Table 2.

**TABLE 2:** Effects of Green tea on reproductive cancers in animal models.

Types	Animal models	Type of green tea	Duration	Positive control	Negative control	Dosage of green tea type/treatment	Outcome measures	Results	Ref
Ovarian Cancer	Subcutaneous human ovarian cancer cells SKOV-3 xenograft in BALB/c nude mice (4–5 weeks old)	EGCG (*n* = 7)	21 days	Paclitaxel (*n* = 7)	Saline (*n* = 7)	10, 30 or 50 mg/kg	Tumor weight: EGCG (reduced by 71.25%, compared to control) and paclitaxel (reduced by 39.62%, compared to control)	EGCG significantly inhibited tumor growth	Qin et al. (2020)
	Subcutaneous human ovarian cancer cells SKOV-3 xenograft in female NCR mice	HA-EGCG (*n* = 10) (intravenous injection)	2 weeks (Twice/week)	Free cisplatin (*n* = 10) and Micellar nanocomplexes (*n* = 10)	An isotonic dextrose solution (*n* = 10)	19.6 mg/kg	Tumor weight: control mice (405.2 ± 141.6 mm^3^); HA-EGCG (No measurement), MNC-treated mice (181.2 ± 75.1 mm^3^)	No significant difference between Hthe A-EGCG and control group	Bae et al. (2017)
Cervical Cancer	Subcutaneous HeLa cells in female athymic nude mice (5/6-week-old)	Nutrient mixture (NM) containing green tea extract (*n* = 12) (diet)	4 weeks	NA	Regular purina mouse chow (*n* = 12)	Nutrient formulation in 20 mg NM	Tumor weight: NM (reduced by 59%; *p* = 0.001, compared to control)	NM group significantly inhibited tumor growth	Roomi et al. (2015b)
	Subcutaneous HeLa cells in female athymic nude mice (5/6-week-old)	Nutrient mixture (NM) containing green tea extract (*n* = 12) (diet)	4 weeks	NA	Regular purina mouse chow (*n* = 12)	Nutrient formulation in 20 mg NM	Tumor weight: NM (reduced by 59%; *p* = 0.001, compared to control)	NM group significantly inhibited tumor growth	Roomi et al. (2015a)
	Subcutaneous CaSki cells xenograft in nude mice	EGCG (*n* = 4) (oral)	30 days	NA	Water (*n* = 4)	35 mM	Tumor volume: Control (22.5 mm^3^) and EGCG (7.5 mm^3^)	EGCG significantly reduced tumor growth and delayed tumor formation	Ahn et al. (2003)
Endometrial Cancer	Subcutaneous human EC cell lines (RL95–2 and AN3 CA) xenografts in nude mice (5–6 weeks old)	ProEGCG (*n* = 5) (oral)	RL95-2 xenograft model: 5 weeks; AN3-CA xenograft model: 3 weeks	NA	Olive oil (*n* = 5)	50 mg/kg	Tumor weight: 1. RL95–2 xenograft model: control (0.55 ± 0.11 g), ProEGCG (0.33 ± 0.16 g); 2. AN3 CA xenograft model: control (1.45 ± 0.59), ProEGCG (0.60 ± 0.32)	ProEGCG significantly inhibited tumor growth and promoted the apoptotic activity of tumors	Man et al. (2020)
	Subcutaneous endometrial cancer cells xenograft in nude mouse	ProEGCG (*n* = 5) (intragastric)	35 days	NA	Olive oil (*n* = 5)	50 mg/kg	No tumor size measurement	ProEGCG significantly inhibited tumor growth and angiogenesis in xenografts	Wang et al. (2018b)
Breast Cancer	Female Sprague–Dawley rat breast cancer carcinogen model (4-week-old)	Green tea powder (*n* = 15) (oral)	9 weeks	NA	Water (*n* = 15)	0.3% (w/v)	Tumor weight: control (8.3 ± 6.9 g), green tea (2.5 ± 4.5 g)	Green tea significantly inhibited tumor growth	Kavanagh et al. (2001)
	Subcutaneous human breast cancer cells MDA-MB231 xenograft in female scid mice (8–10 weeks old)	Green tea extracts (*n* = 12) (oral)	35 days	NA	Water (*n* = 12)	2.5 g/L	No tumor size measurement	GTE significantly inhibited tumor growth and vessel density	Sartippour et al. (2001)
	Breast cancer MDA-MB-231 cells xenograft in female athymic nude mice (6-week-old)	0.5% Nutrient mixture (NM) containing epigallocatechin gallate in regular diet (*n* = 6) (diet)	4 weeks	NA	Regular diet (*n* = 6)	Nutrient formulation	Tumor size: NM (reduced by 27%, *p* = 0.0082, compared to control)	NM significantly inhibited tumor growth	Roomi et al. (2005a)
	Subcutaneous human breast cancer cells MCF-7 xenograft in nude mice	Green tea extracts (oral)	64 days	Tamoxifen	Water	2.5 g/L	Tumor volume: Green tea (341.1 ± 48.8 mm^3^), tamoxifen (177.8 ± 37.6 mm^3^), control (622.2 ± 163.3 mm^3^), dual therapy with green tea and tamoxifen (116.5 ± 31.9 mm^3^)	Green tea and tamoxifen-treated together significantly inhibited growth and promoted apoptosis of tumor	Sartippour et al. (2006)
	Breast cancer MDA-MB-231 cells xenograft in female athymic nude mice (5-week-old)	Green tea polyphenols or EGCG (*n* = 5) (oral)	10 weeks	NA	Water (*n* = 15)	3 mg GTP/1 mg of EGCG	Tumor volume: EGCG (reduced by 45%, compared to control), GTP (reduced by 61%, compared to control)	GTP and EGCG significantly inhibited tumor growth, and proliferation and induced apoptosis of tumors	Thangapazham et al. (2007)
	Heterozygous C3 (1) SV40 T,t antigen (TAg) transgenic female mice (4-week-old)	Green tea catechins (*n* = 16) (oral)	15 weeks	NA	Water (*n* = 16)	0.01 or 0.05% (w/v)	Tumor weights: control (1.93 g), GTC (1.85 g)	GTC significantly inhibited tumor growth	Kaur et al. (2007)
	Subconfluent murine breast cancer cells 4T1 in BALB/c nude mice (6–7 weeks old)	EGCG (*n* = 8) (i.p.)	24 days	Taxol (*n* = 8)	Saline (*n* = 8)	30 mg/kg	Tumor volume: control (800 mm^3^), EGCG (700 mm^3^), Taxol (650 mm^3^), Combo of EGCG and Taxol (250 mm^3^); Tumor weight: control (1.25 g), EGCG (1.15 g), Taxol (1 g), Combo of EGCG and Taxol (0.65 g)	EGCG alone had little effect on tumor growth but the combination of EGCG and taxol significantly inhibited tumor growth	Luo et al. (2010)
	Breast cancer cells SUM-149 xenograft in female NOD/SCID mice (6-week-old)	EGCG (*n* = 6) (i.p.)	42 days	NA	PBS (*n* = 6)	16.5 mg/kg	Tumor volume: EGCG (reduced by 37.7 ± 4.4%, compared to control), tumor weight EGCG (reduced by 28.6 ± 6.5%, compared to control)	EGCG significantly inhibited tumor growth	Mineva et al. (2013)
	Subcutaneous murine breast cancer cells 4T1 in BALB/c nude mice	EGCG (i.p.)	3 days	NA	PBS	10 mg/kg	Tumor volume: Control (8000 mm^3^), EGCG (3700 mm^3^); tumor weight: Control (6 g), EGCG (3.8 g)	EGCG significantly inhibited tumor growth, macrophages infiltration, and M2 polarization of tumors	Jang et al. (2013)
	Subconfluent murine breast cancer cells 4T1 in BALB/c nude mice (5-week-old)	0.5% Nutrient mixture (NM) containing epigallocatechin gallate in regular diet (*n* = 7) (diet)	4 weeks	NA	Regular Purina mouse chow (*n* = 7)	20 mg NM	Tumor weight: NM(0.91 ± 0.43 g), Control (1.83 ± 0.81 g)	NM group significantly inhibited tumor growth	Roomi et al. (2014)
	An orthotopic xenograft mouse model by injecting with early transformed breast cancer SHR cells in nude mice (4–6-week-old)	Green tea polyphenols (Sunphenon 90D) (*n* = 5) (oral)	9 weeks	NA	Regular diet	5 mg/ml	Tumor volume: Control (1200 mm^3^), GTP (300 mm^3^); Tumor weight: Control (1100 mg), GTP (300 mg)	GTPs significantly inhibited tumor growth and the proliferation rate of tumor growth	Li et al. (2016c)
	An orthotopic xenograft mouse model by injecting with MDA-MB-231 cell in nude mice (4–6-week-old)	Green tea polyphenols diet (*n* = 5) (oral)	8 weeks	Tamoxifen (*n* = 5)	Regular diet	3 mg/ml	Tumor volume: Control (1200 mm^3^), GTP (1000 mm^3^), TAM (1200 mm^3^); Tumor weight: Control (1.4 g), GTP (1 g), TAM (1.2 g)	GTPs alone significantly suppressed tumor growth	Li et al. (2017)
	Subcutaneous murine breast cancer cells 4T1 in male BALB/c mice (6-week-old)	Pre-treatment of EGCG (*n* = 5) (oral)	Pre-treatment for 1 month	NA	Water (*n* = 5)	250, 500, 1000 and 2000 μg/ml	Tumor volume: Control (800 mm^3^), EGCG250ug/ml (600 mm^3^), EGCG500ug/ml (400 mm^3^), EGCG 1000 µg/ml (350 mm^3^) EGCG 2000 µg/ml (400 mm^3^)	EGCG in a low concentratioof n 250 μg/ml can significantly inhibit tumor growth	Xu et al. (2020)
Prostate Cancer	Male TRAMP mice (8-week-old)	Green tea polyphenols (oral)	24 weeks	NA	Water	0.1% (v/v)	No tumor size measurement	GTP significantly inhibited tumor growth, inhibited angiogenesis and metastasis in tumor	Adhami et al. (2004)
	Male TRAMP mice (8-week-old)	Green tea polyphenols (oral)	24 weeks (thrice a week)	NA	Water	0.1% (v/v)	No tumor size measurement	GTP significantly inhibited tumor growth	Saleem et al. (2005)
	Inoculation of human prostate cancer cells PC-3 xenograft in male nude mice	Nutrient mixture (NM) containing epigallocatechin gallate in regular diet (diet)	4 weeks	NA	Regular diet	Nutrient formulation	Tumor weight: NM (reduced by 47%, compared to control); Mean tumor value: NM (reduced by 53%, compared to control)	NM significantly inhibited tumor growth, MMP-9 and VEGF secretion, and mitosis in tumor	Roomi et al. (2005b)
	Male TRAMP mice (8-week-old)	Green tea catechins (*n* = 9) (oral)	16 weeks	NA	Water (*n* = 9)	0.3% (w/v)	No tumor size measurement	GTC significantly inhibited tumor growth	Scaltriti et al. (2006)
	Subcutaneous human prostate cancer cells CWR22Rν1 xenografts in male athymic nu/nu mice (6–8-week-old)	Green tea polyphenols (*n* = 10) (oral)	28 days	Celecoxib (*n* = 10)	Water (*n* = 10)	0.1% (v/v)	Tumor growth: GTP (reduced by 42%, compared to control), celexocib (reduced by 57%, compared to control), combination (reduced by 81%, compared to control)	EGCG significantly inhibited the tumor growth, but the combination of EGCG and celexocib had greater effects	Adhami et al. (2007)
	Male TRAMP mice (8-week-old)	Green tea catechins (*n* = 9) (oral)	16 weeks (thrice a week)	NA	Water (*n* = 9)	0.3% (v/v)	No tumor size measurement	GTC significantly inhibited tumor growth	Mccarthy et al. (2007)
	Male TRAMP mice (8-week-old)	EGCG (oral)	23 weeks	NA	Water	0.06% (w/v)	No tumor size measurement	EGCG significantly reduced cell proliferation and induced apoptosis in tumor	Harper et al. (2007)
	subcutaneous CWR22R xenografts in male nude mice (4–5-week-old)	EGCG (*n* = 8), peracetate of EGCG (*n* = 8) (i.p.)	20 days	NA	DMSO (*n* = 8)	EGCG: 50 mg/kg, EGCG-P: 86.7 mg/kg	Tumor size: EGCG (reduced by 36.3%, compared to control) EGCG-P (reduced by 62.9%, compared to control)	EGCG-P had a significantly better therapeutic effect than EGCG and control on inhibiting tumor growth and angiogenesis while promoting apoptosis of tumors	Lee et al. (2008)
	Male TRAMP mice (4-week-old)	Green tea polyphenols (*n* = 20) (oral)	24 weeks (thrice a week)	NA	Water (*n* = 20)	0.1% (v/v)	No tumor size measurement	Green tea polyphenols inducted apoptosis by inhibiting osteopontin and NFκB signaling in TRAMP mice	Siddiqui et al. (2008)
	Subcutaneous human prostate carcinoma cell line LNCaP 104-R1 xenografts in castration male BALB/c nu/nu mice	EGCG (i.p.)	11 weeks	NA	Water	1 mg	Tumor volume: EGCG (reduced by 40%, compared to control)	EGCG significantly inhibited tumor growth and suppressed cell proliferation of tumors	Chuu et al. (2009)
	Male TRAMP mice (8-week-old)	Green tea polyphenols (*n* = 18) (oral)	4 weeks (thrice a week)	NA	Water (*n* = 18)	0.1% (v/v)	Tumor volume and weight: GTP significantly the parameters compared with the control	GTP significantly inhibited cancer development	Adhami et al. (2009)
	Subcutaneous 1 × 106 PC-3 cells in nude mice (6–8 weeks old)	TBS-101 (*n* = 6) (oral)	29 days (once every other day)	Etoposide	5% DMSO in PBS	80 mg/kg	Tumor growth: TBS-101 significantly inhibited	TBS-101 containing green tea significantly inhibited tumor growth and invasion	Evans et al. (2009)
	C57BL/6J and C57BL/6J/Nrf2(−/−) mice	EGCG (*n* = 4) (i.g.)	12 h	NA	PEG 400 aqueous solution	100 mg/kg	No tumor size measurement	EGCG significantly downregulated Nrf2 in tumors	Nair et al. (2010)
	Male TRAMP mice after weaning	Green tea polyphenols (oral)	25 weeks (thrice a week)	NA	Water	0.05% (v/v)	No tumor size measurement	No significant differences between GTP and control	Teichert et al. (2010)
	Human prostate cancer cells 22Rν1 xenografts in male nude mice (6–8-week-old)	EGCG (i.p.)	6 weeks	NA	Water	1 mg	No tumor size measurement	EGCG significantly inhibited tumor growth	Siddiqui et al. (2011)
	Male rat (10-week-old)	Green tea (oral)	4 weeks (over 39 weeks long duration)	Soy diet	Water	2% (v/v)	No tumor size measurement of green tea	Green tea and soy in combination significantly inhibited tumor growth and inflammation	Hsu et al. (2011)
	Subcutaneous 5 × 105 LAPC-4 cells in male SCID mice (fed AIN-93G diet)	Green tea bag (*n* = 12) (oral)	6 weeks (thrice a week)	Q (quercetin)	Water (*n* = 12)	One tea bag in 240 ml of water for 5 min	Tumor size: 0.2% Q (reduced by 3%), 0.4% Q (reduced by 15%), GT (reduced by 21%), GT + 0.2% Q (reduced by 28%) and GT + 0.4% Q (reduced by 45%), all compared to control	Green tea and quercetin in combination significantly improved chemoprevention and suppressed proliferation	Hsu et al. (2011)
	Subcutaneous 1 × 106 22Rv1 cells in 4 weeks old male athymic nude mice	Chit-nanoEGCG (*n* = 12), EGCG (*n* = 12) (oral)	Five times a week, until tumors reached a targeted volume of 1200 mm^3^	NA	Void chitosan nanoparticles (*n* = 12)	Chit-nanoEGCG (3 or 6 mg/kg), EGCG (40 mg/kg)	Times taken to achieve an average tumor volume of 1200 mm^3^: control (32 days), EGCG (46 days), Chit-nanoEGCG (3 mg/kg) (53 days), and Chit-nanoEGCG (6 mg/kg) (60 days)	EGCG significantly inhibited tumor growth, but Chit-nanoEGCG had significantly greater effects	Khan et al. (2014)
	Male TRAMP mice (5-week-old)	Polyphenon E (*n* = 11–16) (oral)	3 weeks (over 25 weeks long duration)	NA	Water (*n* = 11–16)	200, 500, and 1,000 mg/kg	Tumor volume: Control (107.5 ± 60.1 mm^3^), Polyphenono E at 200 mg/kg (90.0 ± 76.9 mm^3^), 500 mg/kg (92.5 ± 53.6 mm^3^), 1000 mg/kg (80.2 ± 36.3 mm^3^)	Polyphenon E significantly inhibited tumor growth and metastasis with no evidence of toxicity	Kim et al. (2014)
	Male Wistar rats (received an intake of cyproterone acetate, testosterone propionate, and N-Nitroso-N-methylurea) (2 months old)	Green tea extract (*n* = 10) (i.g.)	Eight weeks (three times per week)	Exercise training	Water (*n* = 10)	0.1%	Tumor weight: Control (1.84 ± 0.22 g), training (1.38 ± 0.25 g), GTE (0.95 ± 0.08 g), GTE + training (1.03 ± 0.11 g)	GTE significantly inhibited tumor growth	Saedmocheshi et al. (2019)
	Adult male Wistar rats (received an intake of cyproterone acetate, testosterone propionate, and N-Nitroso-N-methylurea) (45 days old)	Green tea extract (*n* = 8) (oral)	8 weeks	Exercise training	Water (*n* = 8)	0.1%	Tumor weight: Control (2.05 ± 0.08 g), training (0.96 ± 0.08 g), GTE (1.38 ± 0.25 g), training+GTE (1.06 ± 0.10 g)	GTE significantly inhibited tumor growth	Vahabzadeh et al. (2020)
	Injection of human prostate cancer cells PC3 cells xenografts through the tail vein in Male NOD-SCID mice (6–8 weeks old)	L-theanine (isolated from green tea leaves) (i.p.)	10 weeks	NA	Saline	80 mg/kg	No tumor size measurement	L-theanine significantly suppressed invasion, migration, and adhesion in tumors	Fan et al. (2021)

EGCG, Epigallocatechin gallate; GTE, green tea extracts; GTP, green tea polyphenols; i.p, intraperitoneal injection; o.g., oral galvage; s.c., subcutaneous injection

These experimental studies demonstrated the ability of EGCG to regulate multi-targets and multi-signaling pathways. Some of these targeted pathways presented potential mechanism of actions of EGCG to regulate autophagy in reproductive cancers. In particular, studies showed that the inhibition of autophagy was conducted *via* inhibiting the MAPK pathway mechanistically (Han et al., 2021; Tam et al., 2021). The Akt/mTor signaling pathway act as the main autophagy regulator for the survival of reproductive cancers (Amaral et al., 2018; Nunez-Olvera et al., 2019; Tam et al., 2021; Wang et al., 2021). Moreover, the NF- κ B pathway not only regulated the cell cycle and apoptosis but also controlled autophagy (Verzella et al., 2020). These will be discussed further in section 4.

### Clinical Trials

Currently, there are very few clinical trials performed to validate whether green tea possesses chemo-preventive or curative activity in significantly reducing cancer development. In clinical trials on prostate cancer, the majority of studies showed significant beneficial effects of green tea extracts on reducing cancer progression than placebo (Bettuzzi et al., 2006; Brausi et al., 2008; Mclarty et al., 2009; Ram Kumar and Schor, 2018). While in breast cancer, the studies by Samavat *et al.* demonstrated the consumption of green tea extracts significantly reduced breast tumors in female patients ((Samavat et al., 2017; Samavat et al., 2019). However, it showed no significant effects when conducted by another study group (Crew et al., 2012; Crew et al., 2015)). These results remained inconsistent owing to the presence of numerous confounding variables in these study designs, such as diet and population differences. In addition, prospective and case-control studies had demonstrated the potential of green tea in reducing the risk of ovarian cancer (Zhang et al., 2002; Yoon et al., 2008; Lee et al., 2013), cervical cancer (Jia et al., 2012; Paul et al., 2019), endometrial cancer (Bandera et al., 2010), breast cancer (Dai et al., 2010; Iwasaki et al., 2010; Iwasaki et al., 2014; Li M. et al., 2016; Yuan et al., 2017), and prostate cancer (Kurahashi et al., 2008; Erdrich et al., 2015; Lassed et al., 2016; Lee et al., 2017; Sawada, 2017; Beynon et al., 2019). A summary of studies based on clinical trials about the anti-cancer effects of EGCG and green tea catechins on reproductive cancers can be found in Table 3.

**TABLE 3:** Effects of Green tea on reproductive cancers in clinical trials.

Type	Study design	Subject	Intervention group	Control group	Length of intervention	Time points	Outcome measures	Results	Ref
Ovarian Cancer	Phase II clinical trial	16	Double-brewed green tea (DBGT; 500 mg epigallocatechin gallate (EGCG)-enriched tea drink)	NA	Minimum <100 days; Maximum 1.5 years	Every 3 months; Endpoint: recurrence starts	Time of recurrence: 5 ppl have an absence of recurrence at 18 months	No significant maintenance in women with advanced-stage ovarian cancer after standard treatment	Trudel et al. (2013)
	Cohort study	244 (Green tea (104), Control (140))	Green tea (2.1 g ± 1.75 g)	Non-tea drinking	Minimum <1 year; Maximum >3 years	Endpoint: death of participants	The survival rate after 3 years: GT 77.9% vs control 47.9%	Green tea enhanced epithelial ovarian cancer survival dose-response relationships	Zhang et al. (2004)
Breast Cancer	Cohort study	390 (≤4 cups/day (181), 5–7 cups (131), and ≥8 cups/day (78))	Green tea (consumption levels of ≤4 cups, 5–7 cups, and ≥8 cups; 30–40 mg EGCG per cup)	NA	6.5 ± 3 years	Endpoint: recurrence starts	Mean expression of ER (fmol/mg protein): ≤4 cups/day (72.8 ± 9), 5–7 cups (93.4 ± 13.8), and ≥8 cups/day (109.7 ± 21.8) (*p* = 0.05); Recurrence Rates: ≤4 cups/day (28.8%), ≥5 cups/day (23.6%) (*p* = 0.05)	Increased consumption of green tea was correlated with decreased recurrence of stage I and II breast cancer	Nakachi et al. (1998)
	Randomized Phase IB Dose Escalation Study	34 (control (8), 800 mg EGCG (14), 1200 mg EGCG (11), 1600 mg EGCG (1))	EGCG (800, 1200, or 1600 mg)	Placebo	6 months	Baseline, 2, 4, and 6 months	No significant changes in serum hormone levels including oestradiol, testosterone, IGF-1, IGFBP-3, and SHBG; target tissue effects including Ki-67 proliferation index or mammographic density; and quality of life	No significant difference in outcome measures between the intervention and placebo group, but this study obtained preliminary data on the biological effects of Poly E to benefit other ongoing trials	Crew et al. (2012)
	Randomized Phase IB Dose Escalation Study	34 (control (8), 800 mg EGCG (14), 1200 mg EGCG (11), 1600 mg EGCG (1))	EGCG (800, 1200, or 1600 mg)	Placebo	6 months	Baseline, 2, 4, and 6 months	Serum HGF levels: Poly E: decreased by 12.7% vs placebo increase of 6.3% (*p* = 0.04), at 2 months, but no statistically significant at 4 and 6 months; serum VEGF levels: Poly E: decreased by 11.5% at 2 months (*p* = 0.02) and 13.9% at 4 months (*p* = 0.05), but no significant difference compared to placebo. Biomarkers of oxidative damage or inflammation, total cholesterol: Poly E treatment: no statistical significance decreased, compared to placebo	No significant difference in outcome measures between intervention and placebo, but this study suggested potential mechanisms of action of tea polyphenols in HGF signaling, angiogenesis, and lipid metabolism	Crew et al. (2015)
	Randomized phase II clinical trial	932 (control (470); GTF (462))	GTE capsule with 328.8 mg total catechins and 210.7 mg EGCG	Placebo	1 year	Baseline and 12 months	Percentage mammographic density of women aged 50–55 years: GTE: reduced by 4.40% vs placebo increase of 1.02% (*p* = 0.05). Overall PMD and modifying effect of COMT genotype: no significant difference in overall	EGCG significantly reduced PMD for women 50–55 years old	Samavat et al. (2017)
	Randomized phase II clinical trial	1075(Placebo (537); GTE-COMT (538))	Green Tea extract-catechol-O-methyltransferase (GTE-COMT)	Placebo	1 year	Baseline, 6 and 12 months	Blood total estradiol and bioavailable estradiol: GTE: increase of 16% (*p* = 0.02) and 21% (*p* = 0.03), compared to placebo at month 12	GTE significantly increased circulating estradiol concentrations and prevented breast cancer	Samavat et al. (2019)
Prostate Cancer	Prospective single-arm clinical trial	19	Green tea extract (500 mg)	NA	Minimum 2 months; Maximum 6 months	Endpoint: A rise of either a relative prostate-specific antigen, or two largest dimensions of measurable disease on CT scan, or intensity of abnormal bone activity of greater than 25% over baseline over a 2-month period	Prostate-specific antigen percentage change: GTE: slow down disease progression in six patients	GTE produced no discernible clinical activity against hormone-refractory prostate cancer	Choan et al. (2005)
	Placebo-controlled study	60 (Placebo (30), GTC (30)	GTC (600 mg)	Placebo	1 year	Baseline, 3, 6, and 12 months	Prevalence of prostate cancer: GTCs: 90% chemoprevention efficacy in men subjected to high risk for developing CaP (*p* < 0.01); Total serum PSA values: GTCs: lower, at any time point, compared to control; Changes in LUTS as assessed by IPSS and QoL scores: GTCs: decrease for 3 months, compared with control (*p* < 0.05))	GTCs bring significant potent chemoprevention activity to cancer patients	Bettuzzi et al. (2006)
	Placebo-controlled study	60 (Placebo (30), GTC (30))	GTC (600 mg)	Placebo	1 year	2 years later following the GTC administration	Incidence of tumors: GTC (3%), placebo (30%); cancer progression onset time: GTC (23.3 months), placebo (19.1 months)	GTC significantly reduced Prostate cancer diagnosis	Brausi et al. (2008)
	single-armed Phase II clinical trial	25	Polyphenon E (EGCG 800 mg)	NA	6 weeks	4, 8 weeks	Serum levels of HGF, VEGF, PSA, IGF-I, and IGFBP-3: EGCG: reduced the biomarkers significantly (all *p* < 0.03))	Polyphenon E played potential roles in prostate cancer treatment or prevention	Mclarty et al. (2009)
	Randomized controlled trial	50 (Placebo (25), GTC (25))	Polyphenon E of 85–95% total catechins, with 56–72% as EGCG	Placebo	3–6 weeks	Baseline, 3 days before surgery, and during surgery	Systemic biomarker 8OHdG/dG, IGF-1, IGFBP-3, Ki67, Caspase 3, microvessel density, PSA value: Polyphenon E group: no significant decrease	Polyphenon E brings no significant differences, suggested longer-term interventions should be used for future studies	Nguyen et al. (2012)
	Randomized controlled trial	97 (Placebo (48), Poly E (49))	Polyphenon E (EGCG 400 mg)	Placebo	1 year	Baseline, 6 and 12 months	Toxicity symptoms, Lower Urinary Tract Symptoms, and Quality of Life scores: Poly E brings significant symptoms unrelated to the study agent, and no other significant difference compared to control	EGCG was safe to be administrated for prostate cancer prevention or other indications	Kumar et al. (2016)
	Single-armed Pre-clinical trial	90	Green tea (10 g w/v))	NA	6 months	Baseline, 3 and 6 months	lipid peroxidation and antioxidants status: GSH (mg/dl): before (20.79 ± 4.32), after 6 months (34.36 ± 3.64), *p* < 0.0001; MDA (nmol/gHb): before (99.52 ± 12.49), after 6 months (45.16 ± 7.45), *p* < 0.0001; CAT (UI/gHb): before (15.29 ± 1.75), after 6 months (22.19 ± 1.78), *p* < 0.0001	Green tea significantly improved overall the antioxidative status in patients	Lassed et al. (2017)

EGCG, Epigallocatechin gallate, GTCs, green tea catechins

## Regulation of Autophagy by EGCG and Green Tea Catechins in Reproductive Cancer

As autophagy is regulated by different autophagy-related proteins that could be potential molecular targets in reproductive cancers. Likewise, EGCG has demonstrated its potential to modulate these factors in current literatures. EGCG restored autophagic flux *via* downregulating Beclin1, Atg5, and p62 in myocardial ischemia/reperfusion injury rats (Xuan and Jian, 2016). Also, EGCG was reported to inhibit dimerization of LC-3BI, promoted LC3B-II production, and activated autophagy in hepatocellular carcinoma cells (Zhao et al., 2017). By promoting the upregulation of Atg5, Beclin-1, and LC3B-II protein levels, EGCG induced apoptosis and autophagy in cisplatin-resistant oral cancer (Yuan et al., 2017). Also, other studies showed that EGCG enhanced LC3 transition and induced Beclin-1in lymphoma (Tsai et al., 2017). Whereas, it regulated Atg5 and Beclin-1 to induce autophagy in subarachnoid hemorrhage (Chen et al., 2018). This regulation of EGCG on the protein level of Atg5 was essentially critical in governing the autophagy and apoptosis switch, as lysosomes would not be able to fuse with phagosome when it was absent (Yousefi et al., 2006). However, the concrete evidence on the modulating effect of EGCG and green tea catechin on autophagy regulation remained controversial. Their role in determining the onset of autophagy might also be dependent on the integration of signaling networks, in which autophagy and cell growth often shared a series of direct or indirect signaling pathways (Neufeld, 2012; Rizzi et al., 2014).

MAPK had been shown to act as a prognostic factor to regulate the incidence of several reproductive cancers ((Eritja et al., 2017; Lim et al., 2019)). EGCG inhibited various transcriptional factors of mitogen-activated protein kinase (MAPK), including c-Jun, and ERK1/2, to inhibit proliferation, and migration and promote apoptosis (Katiyar et al., 2001; Maeda-Yamamoto et al., 2003). As matrix metalloproteinases (MMPs) were correlated with tumor invasion *via* MAPK, MMPs were reduced upon the treatment with EGCG to revoke the activation of ERK and JNK, which prevented fibrosis, invasion, and migration of cancer cells (Kim et al., 2004; Zhen et al., 2006; Ho et al., 2007; Zeng and Harris, 2014). Autophagy also acts as an adaptive resistance mechanism upon the inhibition of MAPK (Lee et al., 2021). MAPK signaling pathway did not solely regulate autophagy (Raufi et al., 2021). The significance of autophagy against MAPK inhibition was also dependent to the cell’s ability to produce autolysosomes (Kochetkova et al., 2017). Autophagic mechanism was activated dependent on MAPK/JNK pathway after therapeutic stress in endometrial cancer (Eritja et al., 2017). In ovarian cancer, the stimulation of tumor cell proliferation *via* the MAPK/ERK1/2/mTor pathway induced autophagy (Lim et al., 2019). Likewise, the promotion of autophagy was found to inhibit tumor cell growth in a breast cancer study *via* the inhibition of MAPK-regulated mTOR activity, and the phosphorylation of ERK was hindered (Sun et al., 2013). Moreover, the downregulation of p-ERK1/2 protein *via* the MAPK/ERK pathway induced early autophagy and apoptosis for anti-tumor activities in breast and cervical cancer (Hanyu et al., 2020; Liu et al., 2020). On the contrary, the promotion of MAPK/ERK pathway was shown to inhibit autophagy on promoting metastasis (Li et al., 2021).

In another aspect, EGCG targeted PI3K/AKT/mTOR pathway *via* inhibiting phosphorylation of AKT and mTOR to exert anti-proliferation and pro-apoptosis effects (Liu et al., 2013; Liu et al., 2016). EGCG also inhibited the binding of EGF to EGFR by reducing receptor dimerization and inhibiting EGFR phosphorylation (Adachi et al., 2007). These regulations indirectly inhibited AKT activities to induce cell cycle arrest (Sah et al., 2004; Hou et al., 2005; Shimizu et al., 2005) As a result, EGCG was shown to increase caspases and anti-apoptotic proteins level, and decrease AKT and pro-apoptotic levels (Moradzadeh et al., 2017). The inhibition of the PI3K/AKT/mTOR pathway promoted autophagy for the anti-proliferation activity in endometrial cancer cells (Lin et al., 2019). The blockade of the AKT/mTOR pathway activated the compensatory MEK/ERK pathway to promote autophagy and apoptosis induced cell death and cell cycle arrest in prostate, cervical and ovarian cancers (Zhou et al., 2015; Xu et al., 2016; Butler et al., 2017; Qin et al., 2018). Another study also showed that the downregulation of p-AKT inhibited cancer cell survival, along with the upregulation of p-JNK1/2-induced apoptosis, synergistically lead to autophagy-induced cell death in the breast cancer cells (Innajak et al., 2016). The promotion of apoptosis and autophagic cell death *via* inhibiting the PI3K/AKT/mTOR signaling pathway could have induced stress to attenuate MAPK/ERK activity in autophagy inhibition (Reddy et al., 2020).

In addition, EGCG also suppressed anti-apoptotic related protein through the inhibition of the NF- κ B pathway (Hastak et al., 2003). EGCG mediated the cleavage of NF- κ B/p65 subunit and deregulated the cell cycle. This further inhibited NF- κ B nuclear translocation and activated caspases for pro-apoptotic activities (Ahmad et al., 2000; Gupta et al., 2004; Kitamura et al., 2012). Typically, It was shown that NF-κB induced autophagy-related genes, including Beclin1 and ATG5, directly trigger autophagy (Salminen et al., 2012). On the other hand, it inhibited autophagocytotic *via* limiting autophagy inducers (including p53 and ROS) or enhancing autophagy repressors (e.g., Bcl-2) (Djavaheri-Mergny et al., 2006; Salminen et al., 2012; Verzella et al., 2020). Hence, the crosstalk between the NF-κB pathway and autophagy in the canner was proposed. Given the mutual upstream regulators of the interplay, therefore, they could control each other *via* positive and negative feedback loops (Trocoli and Djavaheri-Mergny, 2011). The complex interconnections of these pathways were demonstrated in breast cancer as both NF-κB and autophagy regulated cell death. Inhibition of the NF-κB pathway downregulated autophagy-induced DNA damage (Vequaud et al., 2015). However, the inhibition of the NF-κB pathway induced ER stress and autophagy, which showed the potential to regulate both cancer cells and cancer stem cells to avoid tumor recurrence in breast and prostate cancers (Lu C. et al., 2021). Although an enhanced interaction between NF-κB p50 and annexin A4 fucosylation promoted autophagy and progressed ovarian clear cell carcinoma malignancy, the interaction and progression were mainly to be associated with clinical stage and chemotherapy resistance (Wang et al., 2017).

EGCG can work like various chemotherapeutic drugs, which enhanced autophagy-induced cancer cell death (Denton et al., 2015). Currently, EGCG as an autophagy inducer was only studied in breast, cervical, and prostate cancers. The summary on the finding of EGCG and green tea catechins on autophagy is shown in Table 4. In breast cancer, EGCG inhibited tumor growth *in vitro* and *in vivo via* activating autophagy directly, thus regulating glucose metabolism (Wei et al., 2018). Another study in the breast cancer cell line showed that EGCG induced autophagy *via* enhancing autophagosome formation, hence inhibiting proinflammatory mediators HMGB1 (Li et al., 2011). The regulated stages of autophagy in breast cancer were shown to be dependent on the EGCG concentration. EGCG in low concentration upregulated Beclin1, Atg5, and LC3B-II/LC3B-I levels, promoting autophagosomes; However, when EGCG is under high concentration, it would only upregulate Beclin1 and Atg5 to enhance the fusion of the autophagosome with lysosomes to form autolysomes, which resulted in a downregulated LC3B-II/LC3B-I levels. Whereas, high concentrations of EGCG were shown to activate later stages of autophagy in breast cancer cells (Wei et al., 2018).

**TABLE 4:** Green tea polyphenol (EGCG) as autophagy modulator in cancer study from 2011 to 2022 January.

Type of cancer	Compound	Study models	Autophagy modulation	Combination strategy	Ref
**GTP as Autophagy Inducer**					
Breast cancer	EGCG	Cell lines: 4T1 and 4T1	Modulating the levels of the autophagy-related proteins Beclin1, ATG5, and LC3B to induce apoptosis and suppresses glucose metabolism	NA	Wei et al. (2018)
		Mouse xenograft Model			
	EGCG	Cell lines: MDA-MB-361 and MCF-7	Stimulated LC3-II production and autophagosome formation, and inhibited LPS-induced HMGB1 up-regulation and extracellular release	NA	Li et al. (2011)
Cervical cancer	EGCG	Mouse xenograft model	Down-regulated p62 and up-regulated Beclin 1 and LC3 to induce apoptosis	Combined with Doxorubicin in chemo-photothermal synergistic therapy	Chen et al. (2020)
	EGCG palmitate	Cell line: HeLa	Induces autophagosome-lysosome formation for cell death	Encapsulated in ZIF-8 nanoparticles with functionalization of folic acid	Chen et al. (2020)
Prostate cancer	Polyphenon E^®^	Cell lines: PNT1a and PC3	Induces ER stress and unfolded protein response	NA	Rizzi et al. (2014)
	EGCG	Cell line: PC3 cell	Inhibits proteasome activity and induces ER stress	Combined with Bortezomib	Modernelli et al. (2015)
**GTP as Autophagy Inhibitor**					
Cervical cancer	EGCG	Cell lines: HeLa	Induce ROS-mediated lysosomal membrane permeabilization to mediate cell death	NA	Zhang et al. (2012)
Prostate cancer	Tea polyphenols	Cell lines: PC3 and DU145	Activation of the mTOR pathway to induce apoptosis	Combined with Docetaxel	Wang et al. (2018b)

EGCG, epigallocatechin gallate; ER stress, endoplasmic reticulum stress; ROS, reactive oxygen species; VEGF, vascular endothelial growth factors

In cervical cancer, the combination of EGCG and Doxorubicin, which is chemotherapy, was investigated as an autophagy modulator that further improved the therapeutic efficacy of photothermal therapy (Chen et al., 2020). Photothermal therapy solely induced hyperthermia damage to intracellular components and activated autophagy which inhibited apoptotic-induced cancer cell death (Zhang Y. et al., 2018; Li et al., 2019; Chen et al., 2020). However, an autophagy enhancer could suppress inflammation and cancer progression, and therefore promote autophagic-induced cell death. When autophagic flux and the formation of autophagosomes were activated, this would downregulate p62 while upregulating both Beclin1 and LC3 in tumors. Additionally, EGCG also played roles to inhibit the toxins generated from Doxorubicin *in vitro* and *in vivo*, and limited chemo-resistance, resulting in greater efficiency (Shan et al., 2019; Chen et al., 2020)

In prostate cancer, Bortezomib is an effective proteasome inhibitor against tumor growth *via* induction of apoptosis *in vitro* and *in vivo* (Papandreou and Logothetis, 2004), followed by activation of unfolded protein regulator, ER stress, and autophagy (Modernelli et al., 2015; Takenokuchi et al., 2015). The proteasome is mechanistically related to autophagy as both pathways act as protein degradation systems and maintain intracellular proteostasis (Zhu et al., 2010). EGCG potently suppressed proteasome activity in prostate cancer (Kazi et al., 2004), and could potentially induce autophagy as Bortezomib. A study hence showed that co-treatment of Bortezomib with EGCG exacerbated autophagy *via* induction of LC3B transition and an autophagic flux in prostate cancer cells (Modernelli et al., 2015). The therapeutic efficacy of green tea as an autophagy inducer depended on the stage of cancer. Green tea polyphenol activated autophagy in the initial stage of prostate cancer *in vitro*, demonstrated by an increased interconversion of LC3B-I to LC3B-II, which indicated the accumulation of inactivated autophagosomes (Rizzi et al., 2014). However, in advanced stages of prostate cancer *in vitro*, although green tea increased the level of LC3B, there was no change in the interconversion level of LC3B-I to LC3B-II to indicate a net autophagic flux (Mizushima and Yoshimori, 2007; Rizzi et al., 2014).

**FIGURE 2:**
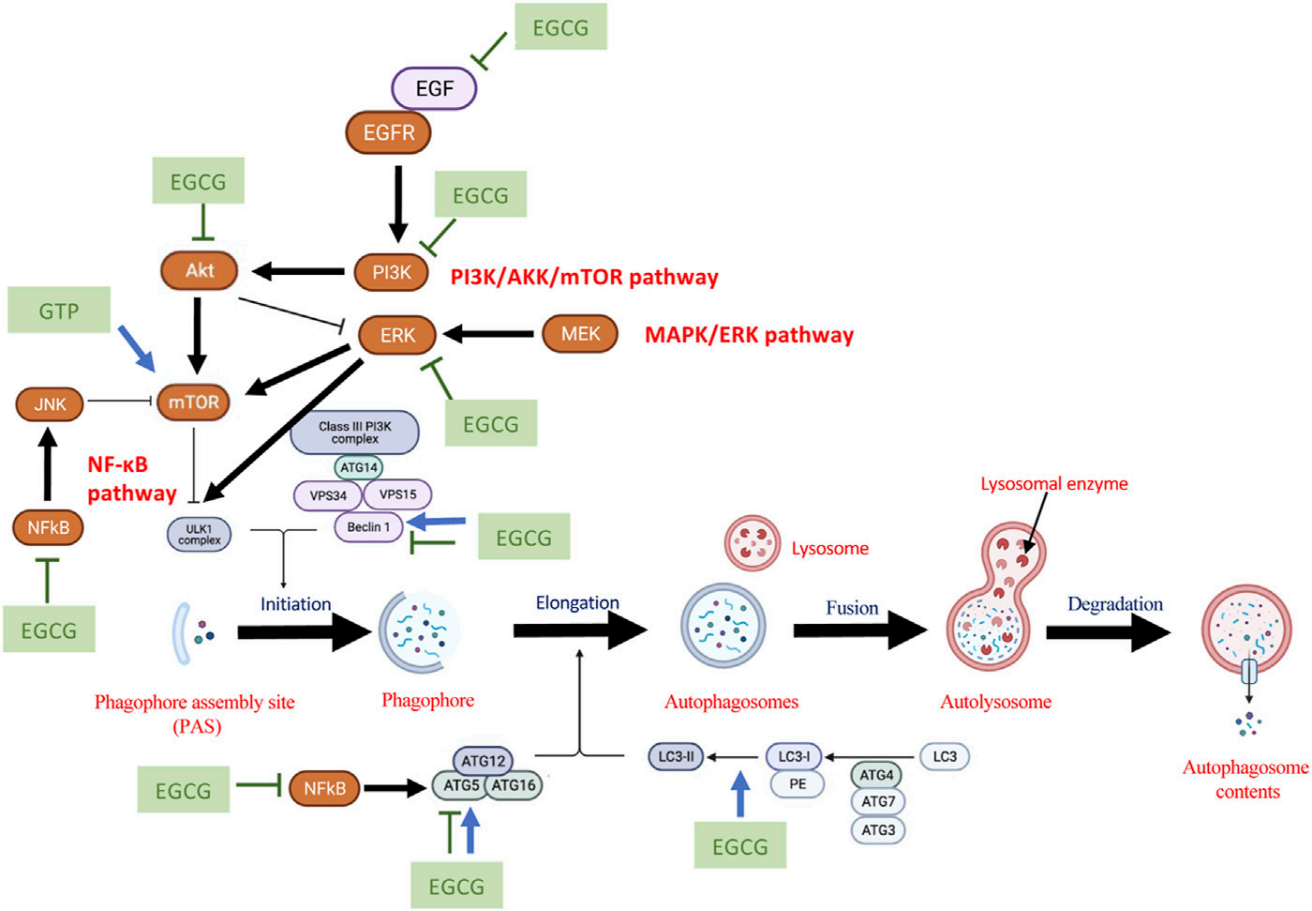
The schematic diagram of potential chemopreventive mechanisms of EGCG and green tea catechins to regulate autophagy. The schematic diagram was created using BioRender.com.

Autophagy degrades unusual macromolecules and organelles that are used to induce apoptosis. An upregulated level of autophagy was shown to limit drug-induced apoptosis in prostate cancer (Herman-Antosiewicz et al., 2006). Induction of autophagy favored cancer cells’ survival under metabolic stress conditions stimulated by chemotherapy or radiation therapy (Ozpolat and Benbrook, 2015). Therefore, a downregulated level of autophagy could enhance the pro-apoptotic properties of chemotherapy. In prostate cancer, patients developed resistance against first-line therapy such as docetaxel (Armstrong and Gao, 2015), which could be due to the induction of autophagy (Wang Q. et al., 2018). Green tea polyphenol inhibited the induced-autophagy events and promoted apoptosis *via* activation of the mTOR signaling pathway. LC3-II was reduced to enhance the p-mTOR expression level (Wang Q. et al., 2018). The schematic diagram of the chemo-preventive mechanisms of EGCG and green tea catechins is shown in Figure 2.

## Potential of EGCG as an Autophagy Modulator in Reproductive Cancer

Pharmacological actions of autophagy modulator are not tumor-selective, which could impose a risk of healthy tissue being transformed into malignant tissue (Gewirtz, 2016; Noguchi et al., 2020). Although autophagy was shown to induce cancer cell death, the anti-oxidative properties of green tea polyphenols inhibited stress-induced autophagy cell death in healthy ovarian granulosa cells. Activities and concentrations of antioxidant SOD and GSH-PX were upregulated. MDA levels, Beclin1, as well as the autophagic vacuoles in cytoplasmic were reduced (Yang et al., 2020). EGCG reduced ROS to limit the enzyme that cleaved LC3-I to form LC3-II, and inhibited LC3-II formation, indicating a reduced level of autophagosome formation and autophagy in healthy ovarian cells (Zhang S. et al., 2020; Yang et al., 2020). Autophagy was induced by environmental stress such as chemicals and toxins (Lee et al., 2011). EGCG shows the potential to regulate autophagy and protect healthy cells against death from chemotherapy.

Reproductive cancer treatments could interfere with the reproductive processes and fertility. EGCG was shown to improve fertility and protect it from reproductive cancers. Its molecular mechanism against infertility is based on regulating ROS, enhancing the antioxidant enzymes’ expression and activity (Roychoudhury et al., 2017; Zhang Y. et al., 2020). From the male fertility aspect, EGCG was able to protect germ cell loss, as well as diminish testes lesions (Ge et al., 2015), sperm deformity (Spinaci et al., 2008), and spermatogenic cell apoptosis (Ge et al., 2015). EGCG in a low concentration improved motility and capacitation of sperm. The addition of EGCG in thawing sperm for *in-vitro* fertilization after chemotherapy significantly increased penetration rate and fertilization efficiency (Rahman et al., 2018). DNA damage on semen from ROS was protected, and the fertility rate was significantly increased. While on the other hand, from the female fertility aspect, EGCG improved developmental capacity and the porcine oocytes’ maturation quality *in vitro*. EGCG in premature cultures of oocytes delayed oxidation and reduced ROS *in vitro* (Roth et al., 2008; Balazi et al., 2019). EGCG inhibitory actions of AMPK, cAMP, calciumion, and ferric iron prevented chromosome impairment, low-density lipoprotein oxidation, and spontaneous mutation. This reduced ROS and regulated gene expression to improve fertility (Rahman et al., 2018).

## Current Limitation and Future Perspectives of EGCG Development

Autophagy modulators can act as tumor activators or suppressors, which complicates the discovery stage in terms of pharmacological, technical, and experimental natures. It must be solved before implement as a chemotherapeutic modulator in the clinic. The autophagy strategy should be customizable and used depending on cancer cell types and tumorigenesis stages. Natural products such as cancer therapies attract growing attention due to their high effectiveness to diminish tumor cells and low toxicity to normal cells (Zhang Q. Y. et al., 2018). Their autophagy roles have been put forward (Rahman et al., 2020; Al-Bari et al., 2021). We reviewed and proposed the use of green tea EGCG as an autophagy modulator in reproductive cancer studies. EGCG exerts extraordinary anti-carcinogenic properties, attributes to its multi-targets and multi-pathways functional properties from anti-proliferative, proapoptotic, anti-invasive, anti-angiogenesis, and anti-oxidative effects (Chacko et al., 2010; Gan et al., 2018; Kochman et al., 2020). It was tested with a safe profile and benefited the preservation of fertility (Rahman et al., 2018; Opuwari and Monsees, 2020), yet it still requires more validation using animal studies and clinical trials to prove the efficacies of EGCG. Fertility-related side effects of EGCG for reproductive cancers in clinical use also needs to be investigated further.

**FIGURE 3:**
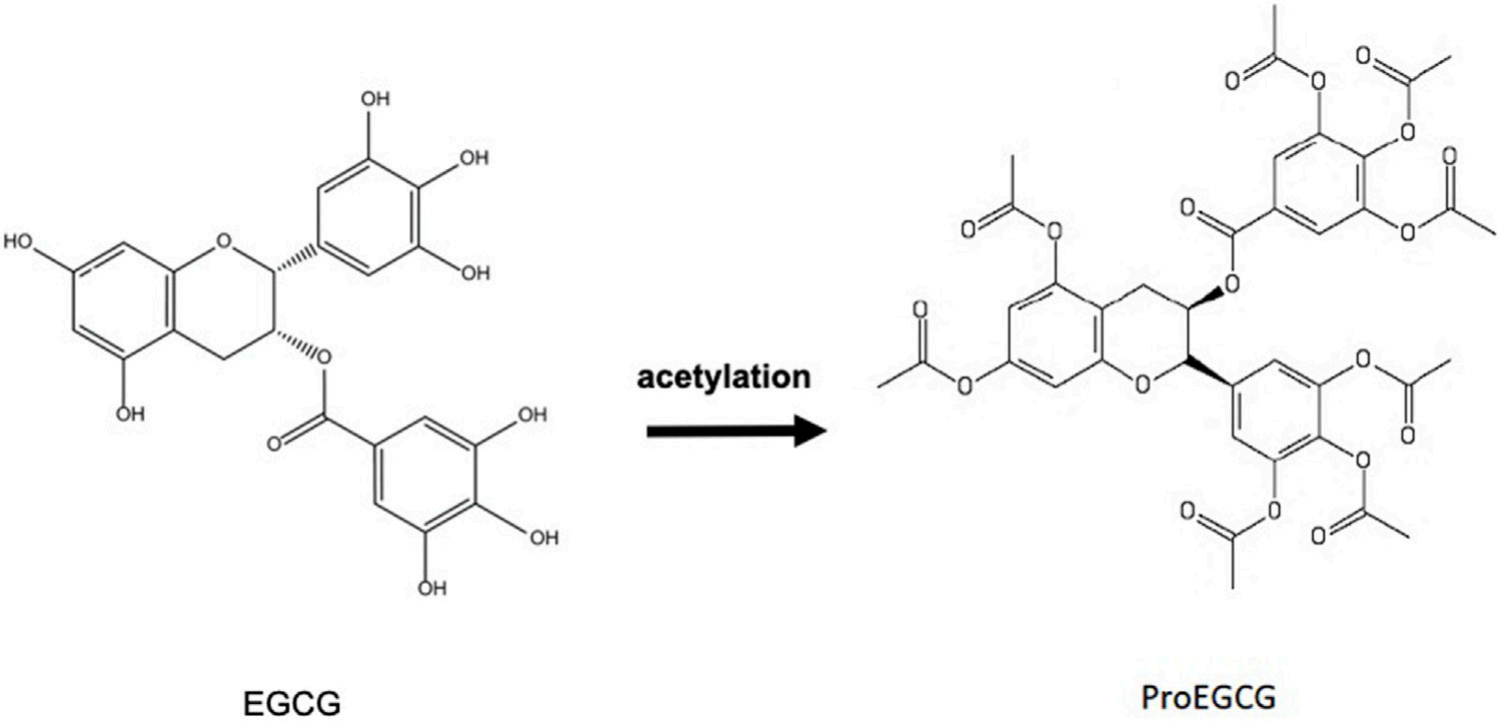
Chemical synthesis of ProEGCG from EGCG.

However, the hydroxyl group on EGCG brings low bioavailability in the human body (Du et al., 2012; Chu et al., 2017). EGCG is not stable and is readily oxidized in a neutral or alkaline environment. A basic environment disfavors the protons on phenol groups, causing it to generate phenoxide anion which acts on electrophilic agents such as free radicals in the body (Lam et al., 2004). With innovations and technologies, this could be overcome *via* different delivery methods including prodrug and encapsulation approaches. Lam et al., team’s derived a prodrug of EGCG (ProEGCG) with enhanced EGCG stability by replacing the -OH hydroxyl groups of EGCG into -COOCH3 acetyl acetoxy groups of ProEGCG (Lam et al., 2004) (Figure 3). ProEGCG showed greater bioavailability and stability than EGCG on breast cancer *in vitro* and *in vivo* (Landis-Piwowar et al., 2005; Landis-Piwowar et al., 2007). Its potency to inhibit tumor growth was to a greater extent than EGCG, with an enhanced inhibitory effect on proteasome and induction abilities of caspase3/7 and PARP proteins, thus to inducing apoptosis. In prostate cancer, ProEGCG limited tumor progression and angiogenesis, as well as induced apoptosis *in vivo* (Lee et al., 2008). Besides these, ProEGCG was shown to enhance the sensitization of leukemia to chemotherapy *via* caspase 3 activity and cleavage of PARP (Davenport et al., 2010). More recently, our team reported that ProEGCG acts as an angiogenesis inhibitor in endometrial cancer models *in vitro* and *in vivo*, and downregulated HIF-1α and VEGFA *via* PI3K/AKT/mTOR pathway (Wang J. et al., 2018). ProEGCG in low doses could inhibit cancer activity in endometrial cancer cells. Based on our finding, it was shown to upregulate the phosphorylation of JNK and p38 MAPK and downregulate that of AKT and ERK to ultimately inhibit tumor cell proliferation, micro-vessels formation and promote apoptotic activity *in vitro* and *in vivo* (Man et al., 2020). Importantly, this anticancer effect was significantlysuperior when compared with EGCG, with no toxicity being found.

On the other hand, a nanostructure-based drug delivery system improves the bioavailability of even low doses of EGCG. Chitosan nanoparticles (Dube et al., 2010; Li et al., 2014), liposomes (Fang et al., 2006; Dag and Oztop, 2017), nanoliposomes (De Pace et al., 2013) encapsulation material were used as the carriers of EGCG in different studies. Improved chemical structure stability *via* sustainable release of bioactive and the enhanced permeability of chemicals through the cellular membrane were resulted (Cai et al., 2018). In breast cancer, EGCG nanoparticles FA-NPS-PEG and FA-PEG-NPS were synthesized. It showed improved cellular uptake and anti-proliferation effect *in vitro* (Zeng et al., 2017). In cervical cancer, EGCG was delivered in a photothermal responsive zeolitic imidazolate framework using polydopamine-PEG nanoparticles (ZIF-PDA-PEG). Infrared laser irradiation assisted the releases of drugs, solving co-loading and multiple drug resistance problems, as well as showed higher efficiency in inducing apoptosis and autophagy (Chen et al., 2020).

Future studies can also focus on combining EGCG with other anticancer drugs to achieve a synergistic enhancement of the anti-cancer effects (Suganuma et al., 2011; Fujiki et al., 2015). In ovarian cancer, green tea with Paclitaxel or other nutrients as combination strategy synergistically inhibited cancer cells (Xinqiang et al., 2017; Panji et al., 2021a). Meanwhile, EGCG in combination with Cruciferous vegetables or Sulforaphane were shown to overcome Cisplatin or Paclitaxel chemotherapy resistance in ovarian cancer (Mazumder et al., 2012; Chen et al., 2013b; a). In cervical cancer, EGCG was treated synergistically with Enoacin or Eugenol-Amarogentin against cancer tumor growth (Pal et al., 2018; Mcdonnell et al., 2019). It was also treated to enhance Cisplatin effects in chemotherapy (Kilic et al., 2015). In breast cancer, EGCG demonstrated synergistic effects with Dexamethasone, U021, Tamoxifen, Quercetin or Paclitaxel (Guo et al., 2006; Sartippour et al., 2006; Li et al., 2009; Luo et al., 2010), thus enhanced cancer cell death. As discussed, tea polyphenol was proposed to be used with other chemotherapies to synergistically advance the therapeutic efficiency of autophagy modulating effect, such as with Docetaxel or Bortezomib in prostate cancer (Modernelli et al., 2015; Wang Q. et al., 2018), with Paclitaxel in ovarian cancer (Panji et al., 2021a), or with Doxorubicin in cervical cancer (Chen et al., 2020).

## Conclusion

The role of autophagy has been shown to play a dual role in cancer. On one hand, autophagy may help to protect against cancer, while on the other hand, it may promote the growth of cancer. As autophagy plays an important role in cell death, these autophagy modulators may be a potential target to induce tumor cell death in a manner independent of necrosis or apoptosis. However, targeting these autophagy modulators as therapeutical strategy remained a huge obstacle. Thus, natural products can be used to target multiple pathways, which provide greater flexibility and potential for a variety of cancer types and stages. Green tea catechins and EGCG have been shown to play an important role in modulating autophagy in reproductive cancer cells. A number of *in vitro* and *in vivo* studies have confirmed the anti-inflammatory and antioxidant properties of polyphenolic compounds to induce autophagic death in reproductive cancer cells. However, it still required more validation using animal studies and clinical trials. Nevertheless, the current evidence strongly suggests the biologically active ingredients of green tea, namely EGCG, to be a valuable and promising agent in reproductive cancer chemoprevention.

## Supplementary Material

Supplementary Figure S1 Chemical structures of the four-major green tea catechins include (−)-epicatechin (EC), (−)-epigallocatechin (EGC), (−)-epicatechin-3-gallate (ECG), and (−)-epigallocatechin-3- gallate (EGCG).
