# Supplementary figures and images for: Green Tea Epigallocatechin-3-Gallate Regulates Autophagy in Male and Female Reproductive Cancer
Source: Front Pharmacol. 2022 Jul 4;13:906746. doi: 10.3389/fphar.2022.906746 (PMC9289441; doi:10.3389/fphar.2022.906746)

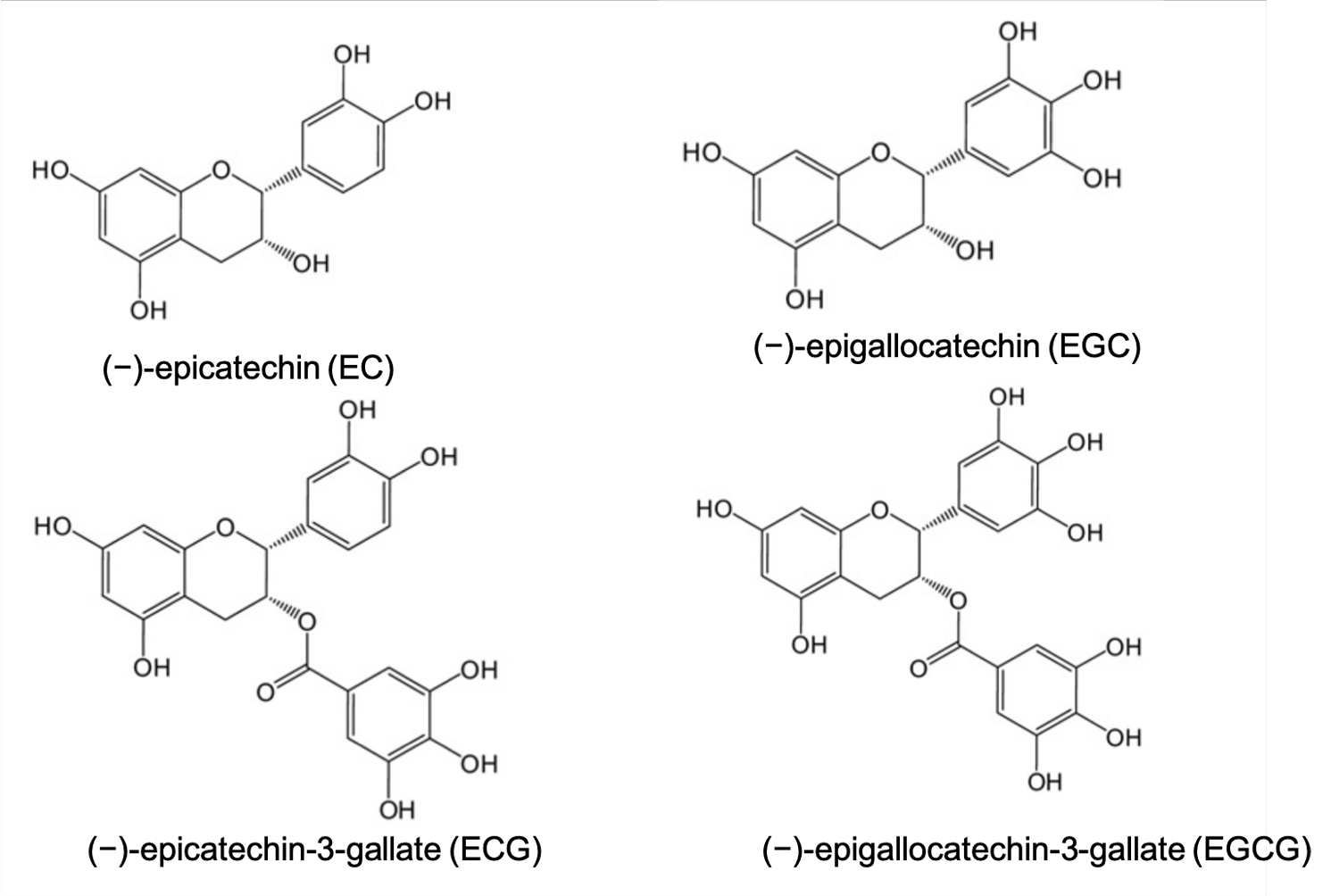

Supplement: Supplementary file 1 [file Image1.tif]
